# Single-cell profiling reveals distinct immune response landscapes in tuberculous pleural effusion and non-TPE

**DOI:** 10.3389/fimmu.2023.1191357

**Published:** 2023-06-26

**Authors:** Xinting Yang, Jun Yan, Yu Xue, Qing Sun, Yun Zhang, Ru Guo, Chaohong Wang, Xuelian Li, Qingtao Liang, Hangyu Wu, Chong Wang, Xinlei Liao, Sibo Long, Maike Zheng, Rongrong Wei, Haoran Zhang, Yi Liu, Nanying Che, Laurence Don Wai Luu, Junhua Pan, Guirong Wang, Yi Wang

**Affiliations:** ^1^ Tuberculosis Department, Beijing Chest Hospital, Capital Medical University, Beijing, China; ^2^ Department of Clinical Laboratory, Beijing Chest Hospital, Capital Medical University, Beijing Tuberculosis and Thoracic Tumor Institute, Beijing, China; ^3^ Department of Emergency, Beijing Chest Hospital, Capital Medical University, Beijing, China; ^4^ National Clinical Laboratory on Tuberculosis, Beijing Key Laboratory for Drug-Resistant Tuberculosis Research, Beijing Chest Hospital, Capital Medical University, Beijing Tuberculosis and Thoracic Tumor Institute, Beijing, China; ^5^ Heart Center, Beijing Chest Hospital, Capital Medical University, Beijing, China; ^6^ Biobank, Beijing Chest Hospital, Capital Medical University, Beijing, China; ^7^ School of Life Sciences, University of Technology Sydney, Sydney, Australia; ^8^ Beijing Chest Hospital, Capital Medical University, Beijing, China; ^9^ Experimental Research Center, Capital Institute of Pediatrics, Beijing, China

**Keywords:** *Mycobacterium tuberculosis*, tuberculosis, tuberculous pleural effusion, ScRNA-seq, local immune response

## Abstract

**Background:**

Tuberculosis (TB) is caused by *Mycobacterium tuberculosis* (*Mtb*) and remains a major health threat worldwide. However, a detailed understanding of the immune cells and inflammatory mediators in *Mtb*-infected tissues is still lacking. Tuberculous pleural effusion (TPE), which is characterized by an influx of immune cells to the pleural space, is thus a suitable platform for dissecting complex tissue responses to *Mtb* infection.

**Methods:**

We employed singe-cell RNA sequencing to 10 pleural fluid (PF) samples from 6 patients with TPE and 4 non-TPEs including 2 samples from patients with TSPE (transudative pleural effusion) and 2 samples with MPE (malignant pleural effusion).

**Result:**

Compared to TSPE and MPE, TPE displayed obvious difference in the abundance of major cell types (e.g., NK, CD4+T, Macrophages), which showed notable associations with disease type. Further analyses revealed that the CD4 lymphocyte population in TPE favored a Th1 and Th17 response. Tumor necrosis factors (TNF)-, and XIAP related factor 1 (XAF1)-pathways induced T cell apoptosis in patients with TPE. Immune exhaustion in NK cells was an important feature in TPE. Myeloid cells in TPE displayed stronger functional capacity for phagocytosis, antigen presentation and IFN-γ response, than TSPE and MPE. Systemic elevation of inflammatory response genes and pro-inflammatory cytokines were mainly driven by macrophages in patients with TPE.

**Conclusion:**

We provide a tissue immune landscape of PF immune cells, and revealed a distinct local immune response in TPE and non-TPE (TSPE and MPE). These findings will improve our understanding of local TB immunopathogenesis and provide potential targets for TB therapy.

## Introduction

Tuberculosis (TB) is caused by *Mycobacterium tuberculosis* and it is one of the leading causes of deaths worldwide. Globally, an estimated 9.9 million people contracted TB in 2020 with 16% corresponding to extrapulmonary forms (1). Tuberculous pleurisy is the second most common form of extrapulmonary TB as well as the main cause of pleural effusion in many countries (1, 2). The pathogenesis of tuberculous pleurisy involves intricate cellular and humoral immune responses (3, 4). Host defense against TB involves infiltration of peripheral blood mononuclear cells (PBMC) into the pleural space (5). This leads to accumulation of immune cells such as lymphocytes and myeloid cells, in the tuberculous pleural fluid (6). As a result, tuberculous pleurisy provides a good model to study the correlates of protective immune responses at the site of infection. However, the mechanism of localized immune response in the pleural fluid remains elusive.

Based on its pathogenesis, pleural effusion can be divided into transudative or exudative pleural effusion. Transudative pleural effusion (TSPE) is caused by systemic factors such as congestive heart failure and liver cirrhosis (7). Exudative pleural effusion is mostly caused by diseased pleural surfaces, such as tuberculous pleural effusion (TPE) and malignant pleural effusion (MPE) (8). Although TPE and MPE are both characterized as lymphocyte-predominant exudates (9, 10), other immune cells such as macrophages, neutrophils and dendritic cells are also present (11). Immune responses dominate depending on different types of pleural effusions. Thus, understanding the heterogeneity, exhaustion, migration and various functional capacity (e.g., effector functions, phagocytosis and antigen presentation) of immune cells in the pleural fluid (PF) from TPE will provide crucial insights into host anti-*Mtb* responses at the tissue level.

Cai Y et al. (12) previously described the local T cell immune landscape in TPE. In Cai’s report, they provide key insights into the spectrum of T cell heterogeneity at TPE. However, TB is a complex inflammatory disease with involvement of various immune cell types besides T cells. Their interactions determine the outcome of TB infection (13). Currently, a comprehensive study into how various immune cells interact and the immune response landscape in *Mtb*-infected tissues (e.g., TPE) is still lacking. Additionally, little is known about the immune features of TPE compared to other pleural effusion like TSPE and MPE.

Single-cell RNA sequencing (scRNA-seq) is a powerful tool for dissecting the immune response and analyzing various cell populations, including cells in complex microenvironments (14, 15). To understand the complex host response to TB and reveal the distinct features among PFs, we performed scRNA-seq to obtain an unbiased and comprehensive visualization of immune responses in pleural fluid mononuclear cells (PFMC) from patients with TSPE, MPE and TPE. Our analysis provides a high-resolution immunological landscape of PFMCs in TPE and reveals distinct response signatures between TPE, TSPE and MPE, facilitating a comprehensive understanding of protective and pathogenic immune responses in patients with TPE.

## Results

### Single-cell transcriptional profiling of pleural fluid mononuclear cells

**Figure 1:**
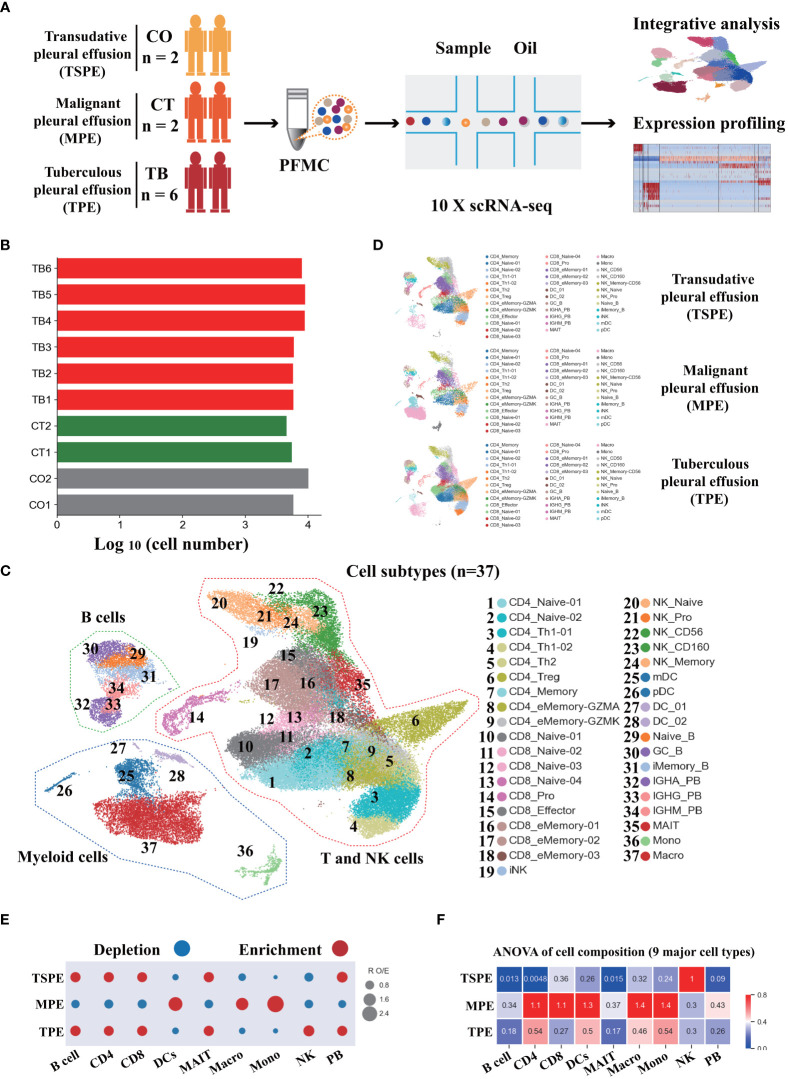
Study design and overall results of single-cell transcriptional profiling of PFs from participants. **(A)** Schematic diagram of the overall study design. 10 subjects, including 2 patients with transudative pleural effusion (TSPE), 2 patients with malignant pleural effusion (MPE) and 6 patients with tuberculous pleural effusion (TPE). **(B)** Bar plot shows the log_10_ transformed cell number of each sample. Red represents the 6 patients with TPE, grey represents the 2 patients with TSPE, and green represents the 2 patients with MPE. **(C)** The clustering result of 37 cell subtypes from 10 individuals. Each point represents one single cell, colored according to cell type. **(D)** The UMAP projection of the 37 cell subtypes in each of the three conditions. Cells are colored by the 37 cell subtypes. **(E)** Disease preference of major cell clusters estimated by R_O/E_. **(F)** Heatmap for q values of ANOVA for disease severity.

Here, we aimed to reveal the immune landscape in pleural fluid (PF) from patients with tuberculous pleural effusion (TPE). We collected fresh PF samples from six patients with TPE before anti-TB treatment (**Figure 1A**). Four fresh PF samples (non-TPE samples), including two samples from patients with malignant pleural effusion (MPE) and two samples from patients with transudative pleural effusion (TSPE), were used for comparative analysis (**Figure 1A**). Thus, the 10 patients were classified into three clinical conditions: tuberculous pleural effusion (TB, *n*=6), transudative pleural effusion (CO, *n*=2) and malignant pleural effusion (CT, *n*=2). The clinical features and laboratory findings of enrolled patients are provided in **Table S1**. We then performed scRNA-seq on these samples (**Figure 1A**). After filtering (see Methods), a total of 70,034 cell transcriptomes was retained across the ten patients, with an average of 5977 unique molecular identifiers (UMIs), representing 2097 genes (**Figures 1B**, **S1**).

Following graph-based clustering of uniform manifold approximation and projection (UMAP), cells were manually annotated based on RNA expression and distribution of canonical cell-type or cell-subtype markers (**Figures S2**, **S3**). We identified nine major cell-types (CD4: CD4+T cells; B: B cells; PB: plasma cells; CD8: CD8+T cells; MAIT: mucosal-associated invariant T cells; NK: natural killer cells; DCs: dendritic cells; Mono: monocytes; Macro: macrophages), and 37 subtypes following sub-clustering. These cells covered various immune cell types in the respiratory system (**Figures 1C, D**, **S2, 3**; **Tables S2–7**). Most of the cell-subtypes were identified in multiple TB patients, suggesting common immune characteristics in TB patients (**Figure S5**).

We determined the compositional changes of major immune cell types in PF. Among PFMCs, 43.25%, 26.58%, 8.8%, 6.55%, 2.45% and 12.28% were CD4, CD8, NK, B, MAIT and myeloid cells (DCs, Mono and Macro), respectively. Compared to TSPE and MPE, multiple immune cells from PFMCs were obviously altered in TPE. We observed a significant decrease of NK cells in TPE relative to TSPE, and a relative expansion of CD4^+^T, B and PB cells (**Figures 1E**, **S4**). In contrast, the relative abundance of DCs, Mono and Macro significantly increased in MPE compared to TPE (**Figures 1E**, **S4**). We also observed a decreased proportion of CD4, B, PB and CD8 in MPE compared to TPE (**Figures 1E**, **S4**). These data indicate that the level of major immune cells in TPE patients (e.g., NK, Mono, Macro) are distinct from non-TPE patients and might be a promising biomarker to diagnose or differentiate TPE from non-TPE.

### Activation of the Th1 and Th17 response as well as T cell apoptosis in patients with TPE

Subtyping indicated a high level of diversity within T cells (CD4, CD8 and MAIT), with 19 different subsets identified (**Figure 1C**, **S3**, **S6**). All T cell subtypes were present in TPE, TSPE and MPE, although the relative percentages varied in a disease-dependent manner (**Figure S6**). Among the 19 different subtypes, we defined 9 subtypes of CD4 T cells, 9 subtypes of CD8 T cells, and an additional cluster of MAIT cells (**Figures S6A, B**; **Tables S3, 4**). We next defined CD4 T and CD8 T subsets according to their expression of classical subtype-specific marker genes and subtype-specific gene expression patterns (**Table S3**; **Figures S2B, C**, **S3A, B**). For CD4 T cells, we annotated two naïve CD4 T cell subtypes (CD4_Naïve-01 and CD4_Naïve-02), one CD4_Memory subset (CD4_Memory), two Th1 subtypes (CD4_Th1-01 and CD4_Th1-02), two effector memory subsets (CD4_eMemory-GZMA and CD4_eMemory-GZMK), one CD4 regulatory subtype and one Th2 subtypes (CD4_Treg) (**Figures S2B**, **S7A**, and **Table S3**). We observed a relative expansion of four CD4 T subsets (CD4_Naïve-01, CD4_Naïve-02, CD4_Memory and CD4_Th1-02) in patients with TPE compared with patients with TPE and MPE, while a decreased proportion of CD4_Th1-01 and CD_eMemory-GZMA were found in patients with TPE (**Figure S6**). Furthermore, CD4 T cells in patients with TPE were enriched with activation genes such as CD69 and IFNG (**Figures S7A, S7C**). Likewise, we also annotated 9 subtypes of CD8 T cells, including four naïve CD8 T cell subclusters (CD8_Naïve-01, CD8_Naïve-02, CD8_Naïve-03 and CD8_Naïve-04), one proliferative CD8 T subclusters (CD8_Pro) and 3 effector memory subclusters (CD8_eMemory-01, CD8_eMemory-02 and CD8_eMemory-03) (**Figures 1D**, **S2C**, **S3B**, **S7B**; **Table S4**). Particularly, CD8 T cell types in patients with TPE were enriched with activation gene CD69 (**Figures S7B, C**).

**Figure 2:**
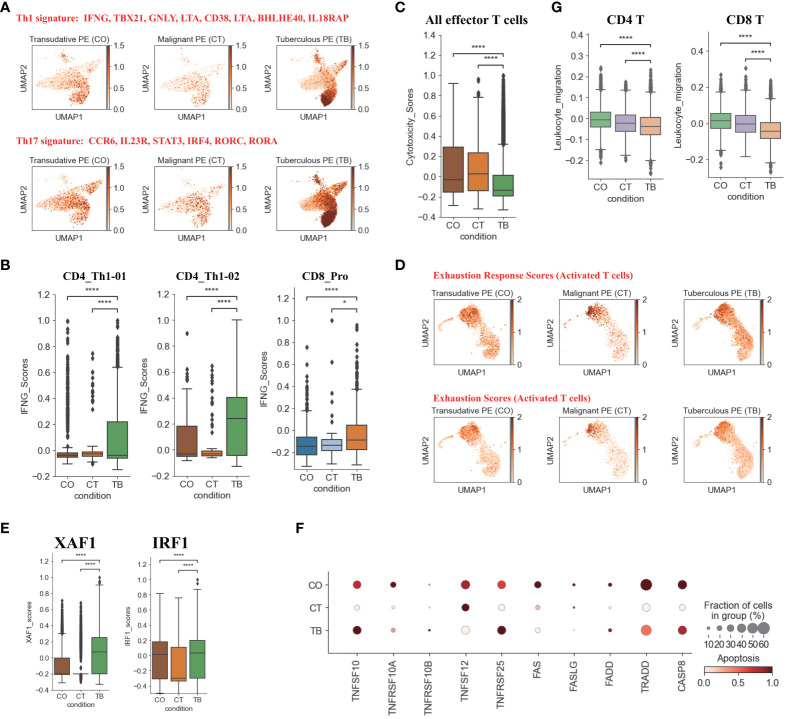
Characterization of gene expression differences in CD4^+^ and CD8^+^T cells across three conditions. **(A)** UMAP plots of mean gene expression from Th1 (Top) and Th17 (Bottom) gene signatures, split by condition. **(B)** Box plots showing the IFNG expression in CD4_Th1-01, CD4_Th1-02 and CD8_Pro subset per condition. **(C)** Box plots showing the cytotoxicity scores in effector T cells across different conditions. **(D)** UMAP plots of exhaustion response scores and exhaustion scores in activated T cells, split by condition. **(E)** Box plots showing XAF1 and IRF1 expression in T cells across each condition. **(F)** Dot plots showing the expression of selected apoptosis-associated genes in T cells across each condition. **(G)** Box plots of leukocyte migration scores in T cells across different conditions. Student’s T-test was applied to test significance in **(B,C, E, G)** *p<0.05, ****p<0.0001.

Among the CD4 T cell subtypes, Th1 cells (CD4_Th1-01 and CD4_Th1-01) are thought to play crucial role in combating *Mtb* infection by secreting important cytokines (e.g., IFN-γ and TNF). We observed that CD4 T cells were enriched in Th1 gene signatures (e.g., TBX21, GNLY, BHLHE40, IFNG) in patients with TPE (**Figure 2A**). Consistently, we also found that the expression of IFN-γ in Th1 cells was significantly higher in patients with TPE than in patients with TSPE and MPE (**Figure 2B**). This implies that Th1 cells in TPE are capable of producing high levels of IFN-γ. These data suggest that the CD4 T cell population in TPE was skewed towards a Th1 response, which is consistent with previous reports (16, 17). Additionally, a CD8 T cell subtype (CD8_Pro) displayed significantly higher expression of IFN-γ in patients with TPE (**Figure 2B**), suggesting that proliferative CD8 T cells might be another source of IFN-γ in TB patients. Besides IFN-γ, TNF also plays an import role in granuloma formation and controlling *Mtb* infection by generating reactive nitrogen intermediates together with IFN-γ (18). Therefore, this study also examined its expression in Th1 cells but it was not significantly upregulated in patients with TPE compared to patients with TSPE and MPE (**Figure S7D**). In addition, CD4 T cells in patients with TPE were enriched in Th17 signature gene such as CCR6, RORA, RORC, IRF4, STAT3 and IL23R, indicating activation of the Th17 response (**Figure 2A**). The above results suggest that CD4 population in TPE favored a Th1 and Th17 response.

In addition to the production of cytokines, T cells, especially effector T cells, can release cytotoxic molecules (e.g., perforins, granzymes) to directly kill *Mtb*, cause apoptosis of target cells and lead to immunopathology (19). Therefore, we used a cytotoxicity score to evaluate the cytotoxic state of each effector T cell subtype across three conditions. Patients with TPE had the lowest cytotoxicity sores in the effector T cell subsets (**Figure 2C**) whereas patients with transudative PE had the highest cytotoxicity score in the effector CD 8 T cell subsets. For patients with malignant PE, they had the highest cytotoxicity score for effector CD4 T cell subsets (**Figure S7E**). Consistent with these results, patients with TPE displayed lower expression of cytotoxic genes than patients with transudative and malignant PE, with the exception of GNLY (**Figure S7F**). These results indicate that effector T cells from patients with TPE might have lower cytotoxicity.

In *Mtb* infection, CD4 and CD8 T cells are exposed to persistent *Mtb* antigens, and this scenario might lead to deterioration of CD4 and CD8 T cell function: a state named “exhaustion”. Thus, we tested whether TPE patients with exposure to persistent *Mtb* antigen had exhaustion in CD4 and CD8 T cells. According to the expression of exhaustion response genes and exhaustion markers, we defined an exhaustion response score and used this score to evaluate the exhaustion state of each activated T cell subset. Our scRNA-seq analysis suggested that, at the bulk level, activated T cells in TPE did not exhibit higher exhaustion scores compared to TSPE and MPE (**Figure 2D**). We also did not observe any exhausted T cells in PF from TPE (**Figure S8A**). In addition, we also did not find that activated T cells in PF from TPE highly expressed typical inhibitory molecules (e.g., PDCD1, LAG3, HAVCR2) (**Figure S8B**). These suggest that CD4 and CD8 T cells in PF from TPE might not undergo exhaustion.

Apoptosis is an important component of pathogen-induced cell death (20). We next investigated the expression of genes in apoptosis-related XAF1, TNF and FAS pathways. XAF1 is involved in pro-apoptotic responses and forms a positive feedback loop with IRF1 to initiate cell apoptosis under stress (21). Through post-translational modification, XAF1 is able to enhance TP53-mediated cell apoptosis (22). The expression of genes related to XAF1-mediated cell apoptosis, including XAF1, IRF1, TP53, BCL2L11, and CASP3, were investigated (**Figures S8C, D**). The expression of XAF1 and IRF1 were significantly increased in T cells from patients with TPE compared to patients with TSPE and MPE (**Figure 2E**). Expression of XAF1 was increased in all T cell subsets in patients with TPE, while IRF1, TP53, BCL2L11, and CASP3 displayed different patterns in different T cell subsets (**Figures S8C, D**). In addition to the XAF1-mediated apoptosis pathway, the expression of genes in other apoptosis-associated pathways, including TNF- and Fas-mediated apoptosis, were also analyzed in T cells. The expression of TNFSF10 and its receptor TNFRSF10B were upregulated in T cells from patients with TPE relative to patients with TSPE and MPE (**Figure 2F**). Another TNF pathway gene, TNFRSF25, was also increased in T cells of patients with TPE. For the FAS pathway, the expression of FAS, FASLG, FADD, TRADD and CASP8 were notably decreased in T cells of patients with TPE (**Figure 2F**). Taken together, these results support the hypothesis that patients with TPE might have increased T-cell apoptosis due to upregulated genes associated with the XAF1- and TNF-apoptosis pathways.

We also examined the migration state of T cells in patients with TPE using a migration scoring system (**Figure 2G**). T cells in patients with TPE did not exhibit a stronger migration score compared to patients with TSPE and MPE. In contrast, T cells from patients with TSPE likely underwent migration as they had the highest migration score (**Figure 2G**).

### NK cell exhaustion observed in patients with TPE

**Figure 3:**
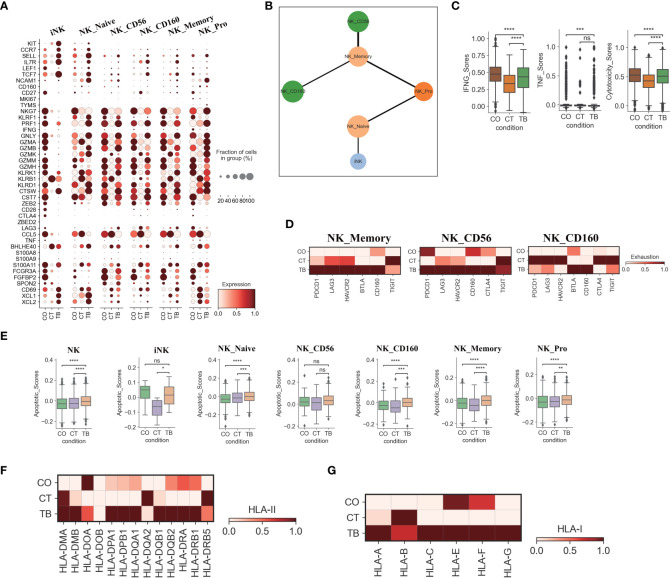
Characterization of gene expression differences in NK cells across three conditions. **(A)** Dot plots showing the expression of selected genes in each NK cell subtype per condition. **(B)** PAGA analysis of NK cell pseudo-time: the associated cell type is shown. **(C)** Box plots of the expression of IFNG, TNF and cytotoxicity scores in NK cells per condition. **(D)** Heatmap plots of the expression of selected exhaustion associated genes in NK_memory, NK_CD56 and NK_CD160 cells per condition. **(E)** Box plots showing the apoptotic scores in NK cells and subtypes per condition. **(F)** Heatmap plots of HLA-II molecules in NK cells per condition. **(G)** Heatmap plots of HLA-I molecules in NK cells per condition. Student’s T-test was applied to test significance in **(A-E)**. *p<0.05, **p<0.01, ***p<0.001, ****p<0.0001, ^ns^p>0.05.

Six NK cell subclusters were observed in our scRNA-data including immature NK cells (iNK), naïve NK (NK_naïve), NK_CD56, NK_CD160, memory NK (NK_Memory-CD56) and proliferative NK cells. (**Figures 1D**, **S2D**, **S3C**, **S9**). Besides immature NK cells, the other five NK subclusters in patients with TPE showed high expression of activation and/or cytotoxic genes. This includes naïve NK cells (GNLY, GZMB, PRF1, KLRD1, CTSW), NK_CD56 (LAG3, BHLHE40, S100A11 and CTSW), NK_CD160 (PRF1, GNLY, GZMK, KLRB1, CTSW, CST7), memory NK cells (LAG3, KLRK1, S100A11, CTSW, KLRD1, KLRK1) and NK_Pro (GNLY, GZMA, GZMB, NKG7, CTSW, etc.) (**Figures 1D**, **3A**, **S2D**, **S9**; **Table S5**).These data indicate the presence of an activated NK cell response as a distinct feature in patients with TPE. Three NK cell subclusters (iNK, NK_naïve and NK_Pro) had an increased trend in patients with TPE, while a decrease trend was observed for NK_CD56, NK_CD160 and NK_Memory-CD56 (**Figure S9F**). In addition, we found that NK cells from patients with TPE had high expression of tissue-resident NK (rNK) cell markers such as CD69, CXL1 and XCL2, but low expression of circulating NK cell markers (cNK) (FCGR3A, FGFBP2 and SPON2) (**Figure 3A**). In contrast, NK cells from transudative PE had high expression of cNK markers (**Figures 3A**, **S10A**). This suggests a predominance of rNK cells in TPE and a predominance of cNK cells in transudative PE.

We then applied PAGA (partition-based graph abstraction) to analyze the global connectivity and potential trajectory topology in the NK cell state transitions. Our data revealed that several nodes showed the high connectivity between NK cell subclusters, implying that these nodes represent potential trans-differentiation bridges (**Figure 3B**). The proliferative NK subcluster (NK_Pro) seemed to be an intermediate state, which connected immature and naïve NK cells to all other subclusters (NK_Memory-CD56, NK_CD56 and NK_CD160). In addition, we also observed high connectivity between NK_Memory-CD56 and NK_CD56 and between NK_Memory-CD56 and NK_CD160 (**Figure 3B**). This suggests that NK_Pro might serve as an intermediate subcluster, which could be valuable for therapeutic strategies targeting this intermediate state.

Similar to Th1 cells, the NK cells also can produce anti-*Mtb*-associated cytokines (e.g., IFN-γ and TNF). IFN-γ and TNF in NK cells were significantly downregulated in TPE comparing to TSPE, but upregulated relative to MPE (**Figure 3C**). Furthermore, NK cells, which contribute to anti-*Mtb* host defense through cell-related cytotoxicity, exhibited lower cytotoxicity scores in TPE compared to TSPE, with the lowest cytotoxic score in MPE (**Figure S10B**). These data results suggest that lower levels of IFN-γ, TNF and cytotoxicity scores in NK cells from patients with TPE may lead to ineffective immune response to *Mtb* infection. Furthermore, the dysfunctional NK response in patients with TPE and MPE might be related to immune exhaustion, and thus we sought to explore the potential sources of NK exhaustion in patients with TPE.

We observed a significant increase in expression of exhaustion response genes and exhaustion markers in NK_Memory, NK_CD56 and NK_CD160 cells from patients with TPE (**Figure S10C**). This includes high expression of multiple inhibitory receptors such as PDCD1, LAG3, HAVCR2, BTLA, CD160, CTLA4 and TIGIT (**Figure 3D**). In contrast, patients with TSPE displayed the lowest exhaustion response scores and exhaustion scores in these three NK subclusters (**Figure S10C**). These results indicate that NK_Memory, NK_CD56 and NK_160 cells might be functionally impaired in patients with TPE.

We also further investigated the apoptosis and migration of NK cells. Significant activation of apoptosis pathways were observed in NK cells from TPE, with four subsets (NK_Naive, NK_CD160, NK_Memory and NK_Pro) exhibiting higher apoptotic scores in patients with TPE than patients with TSPE and MPE (**Figure 3E**). Genes associated with the XAF1-, TNF- and Fas-apoptosis pathways (e.g., TNFSF10, FADD, XAF1 and CASP8) were upregulated in NK cells from patients with TPE, suggesting that these pathways might cause the increased NK cell apoptosis observed in patients with TPE. This study did not find significant activation of NK migration in TPE relative to TSPE and MPE (**Figure S10E**), implying that NK cells in TPE did not undergo migration. In addition, we observed that genes encoding HLA class II molecules (e.g., HLA-DMB, HLA-DPA1, HLA-DPB1) and HLA Class I (HLA-A, HLA-C and HLA-G) were highly expressed in NK cells from patients with TPE compared to patients with transudative and malignant PE (**Figures 3F, G**). The upregulation of HLA class II molecules is important for various pathways (e.g., promote crosstalk between NK cells and DCs).

### Features of myeloid cells in patients with TPE

**Figure 4:**
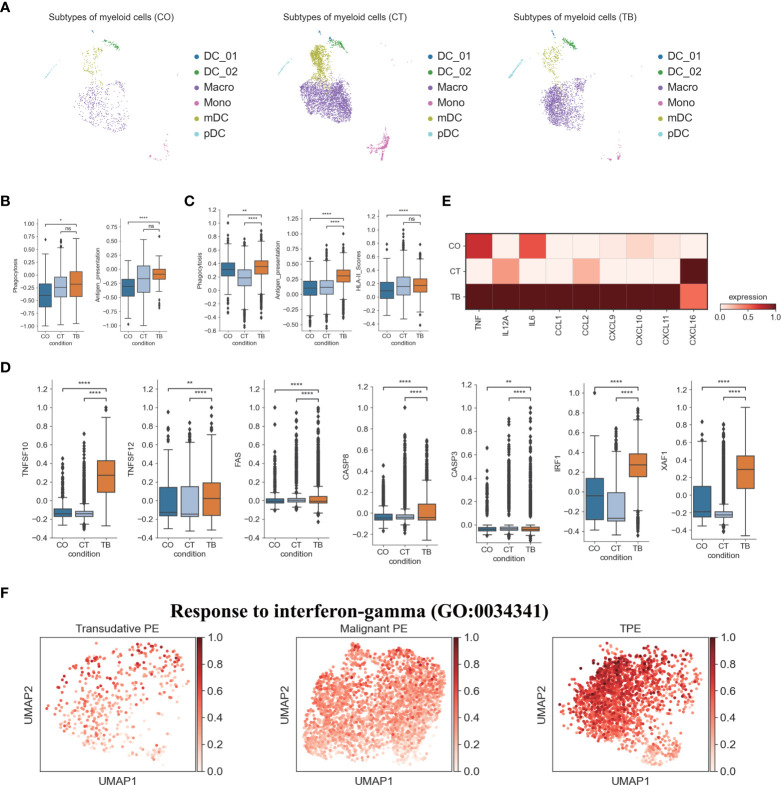
Characterization of gene expression differences in myeloid cells across three conditions. **(A)** The UMAP projection of the 6 myeloid cell subtypes across three conditions. Cells are colored by the 6 myeloid cell subtypes. **(B)** Box plots showing the phagocytosis and antigen presentation scores in monocytes across three conditions. **(C)** Box plots showing the phagocytosis, antigen presentation and HLA-II molecule scores in macrophages across three conditions. **(D)** Box plots of the expression of apoptosis-related genes in macrophages across three conditions. **(E)** Heatmap showing the expression of selected genes in macrophages across three conditions. **(F)** UMAP plots showing mean gene expression of interferon-gamma response gene signatures in macrophages, split by condition. Student’s T-test was applied to test significance in **(B–D)**. *p<0.05, **p<0.01, ****p<0.0001, ^ns^p>0.05.

Transcriptome analysis of myeloid cells identified 4 DC subsets, 1 monocyte subset and 1 macrophage subset (**Figures 4A**, **1D**, **S11B**, **S11**; **Table S6**). pDCs play an important role in microbial sensing and secrete type I interferons (IFNs) in response to microbial infection (4). Our scRNA-seq analysis found that pDCs from TPE highly expressed microbial recognition receptors like TLR7 and TLR9, and interferon production-related genes such as IRF1, IRF7, IRF8, PACSIN1 and DERL3 (**Figure S11B**). IRF1 and IRF5 are important for expression of type I IFNs in DCs and had high expression in pDCs from TPE (**Figure S11B**). CCR7, as a key chemokine receptor in pDCs, is upregulated upon exposure to TLR ligands, and we observed increased expression of CCR7 in TEP relative to transudative and malignant PE (**Figure S11B**). In addition, the percentage of pDCs was significantly increased in patients with TPE compared to patients with TSPE and MPE (**Figure S11**). These results suggests that pDCs in TPE may be involved in anti-*Mtb* response.

In contrast, the percentage of mDCs was significantly decreased in TPE relative to TSPE and MPE, and comprised of ~10% of all myeloid cells in TPE (**Figure S11**). However, mDC from TPE had relative low expression of the Fc epsilon receptor gene FCER1A. This may reflect variation in mDC states between different PFs. mDC, which specializes in antigen processing, play a crucial role in the interface between innate and adaptive immunity. Our analysis suggests that mDCs from TPE had a relatively high expression of HLA-DR molecules, which are essential for antigen presentation (**Figure S11B**). In addition, the high expression of these HLA class II genes validated that the mDCs cluster was activated. In addition, genes involved in mDCs development such as RELB, RBPJ, IRF2 and IRF4, were highly expressed in mDCs from TPE (**Figure S11B**). Genes associated with neutrophil activation were expressed at high levels in mDCs from TPE, while genes related to the proinflammatory response such as CCL3, CCL5 and CXCL8 were expressed at lower levels in mDCs from TPE compared to TSPE and MPE (**Figure S11B**). These data showed that mDCs from TPE might also have a positive role in anti-*Mtb*, and a minor contribution to the proinflammatory response. In addition to mDC and pDC, we observed two subsets corresponding to DC1 (DC_01: CLEC9A, CADM1, CAMK2D) and DC5 (DC_02: LYZ, PPP1R14A) dendritic cell types defined previously (**Figures 1**, **S11**) (23). Similar to the results observed in mDCs, DC_01 and DC_02 had a relatively high expression genes associated with HLA-DR molecules, neutrophil activation and DCs development (**Figure S11B**), implying that these DC subsets may also contribute to anti-*Mtb*.

We also investigated TPE-related differences in monocyte composition. Comparing the relative cell proportions in patients with TSPE and MPE, we observed a notable decrease in monocytes in patients with TPE (**Figure S11**). This cluster highly expressed S100A family genes (e.g., S100A8, S100A9) in patients with TPE and MPE, which are characteristic markers of human myeloid-derived suppressor cells (24). This suggests that monocytes may contribute to immune paralysis in TB and tumor patients (**Figure S11B**). In addition, monocytes from TPE had relatively high expression of cell proliferation genes (e.g., EIF5A), IFN-inducible genes (e.g., ISG15, MX1, MX2), antigen presenting genes (e.g., HLA-DRA, HLA-DQA1, HLA-DPB1, CD74) and component 1q genes (e.g., C1QA, C1QB and C1QC) (**Figure S11B**), suggesting that monocytes might play an important role in anti-TB infection. In particular, monocytes from TPE indicated greater functional capacity, including phagocytosis and antigen presentation, than TSPE and TPE (**Figures 4B**, **S12A**), as evidenced by the high expression of phagocytosis-related genes and HLA-II components (**Figure S12A**).

We also identified a macrophage subset (Macro: LYZ, CST3, CD68, CD163) (**Figures 1D**, **S11B**), which had a notable increase in patients with TPE relative to patients with TSPE and MPE (**Figure S11**). Macrophages can engulf *Mtb* through a series of membrane invagination, budding and fusion events, leading to the formation of the phagosome (25). Thus, we first analyzed the phagocytosis capacity of macrophages, and found that macrophages in patients with TPE indicated greater phagocytosis capacity than patients with TSPE and MPE (**Figure 4C**). We observed significant increased expression of phagocytosis-associated genes (e.g., ARF6, RAC2, PRKCE, VAV2) in macrophages from TPE (**Figure S12B**). After phagocytosis, macrophages can deliver these materials for antigen processing and presentation to activate T cells and the adaptive immune response against *Mtb*. Hence, we also investigated the antigen presentation capacity of macrophages, and observed that macrophages from TPE exhibited higher antigen presentation capacity than TSPE and MPE (**Figure 4C**). Compared to TSPE and MPE, macrophages from TPE highly expressed antigen presentation-associated genes (e.g., CITA, RFX5, B2M, HLA-F, HLA-DQA2, TAP1) (**Figure S12C**). MHC class II molecules, which play an important role in antigen presentation, were significantly upregulated in macrophages from TPE relative to TSPE and MPE (**Figures 4C**, **S12C**). Furthermore, macrophage apoptosis, which releases apoptotic vesicles carrying *Mtb* antigens to *Mtb*-uninfected DCs, can result in more effective antigen presentation (26). Genes associated with the TNF-, Fas- and XAF1-apoptosis pathways (e.g., TNFSF10, TNFSF12, FAS, CASP8, XAF1) were upregulated in macrophages from patients with TPE, suggesting an increase in macrophage apoptosis in patients with TPE (**Figures 4D**, **S12E**). In addition, the activation of macrophages can result in secretion and production of various cytokines and chemokines, which attracts NK, T cells, neutrophils, and more DC and macrophages to the *Mtb*-infection site. Genes encoding cytokines and chemokines (TNF, CCL1, CCL2, CXCL9, etc) were upregulated in patients with TPE compared to patients with transudative and malignant PE (**Figure 4E**). To further examine the anti-*Mtb* immune responses of macrophages, we also investigated the expression of genes belonging to the Gene Ontology (GO) biological process term: response to interferon (IFN)-gamma in macrophages. We found that response to IFN-γ was significantly upregulated in macrophages from TPE compared to transudative and malignant PE (**Figures 4F**, **S12D**). These results indicate that macrophages in patients with TPE displayed strong anti-*Mtb* response.

### Features of B cells in patients with TPE

A comprehensive analysis of both cellular and humoral immunity could contribute to a better understanding of the immune response to TB. Currently, less is known about the role of B cell-mediated immunity in protection against *Mtb*-infection. Therefore, we analyzed the scRNA-seq result of B cells in the immune response to *Mtb*. A total of 6 B cell subclusters were identified according to classical B cell markers including Naïve B cell (Naïve_B), Germinal center B cell (GB_B), Intermediate transition Memory B cell (iMemory_B), IGHA expressing plasma cell (IGHA_PB), IGHM expressing plasma cell (IGHM_PB) and IGHB expressing plasma cell (IGHG_PB) (**Figures 1**, **S2**, **S13**; **Table S7**). We then examined the compositional changes of the 6 categories of B cells in PE. Naïve_B, GB_B and iMemory_B did not show significant changes among patients with TPE, TSPE and MPE (**Figure S13**). However, plasma cell clusters may be associated with different PE conditions. Using pseudo-time analysis, we observed that plasma cells appeared to be derived from memory state B cell (iMemory_B) (**Figure S14A**). The percentage of IGHM_PB and IGHG_PB reached ~15% and showed an increased trend in patients with TPE (**Figure S13**). In contrast, for IGHA-PB, it was highest in patients with malignant PE, reaching ~20% (**Figure S13**). These data suggest that increased IGHM and IGHG plasma cells appears to be a feature of TPE.

**Figure 5:**
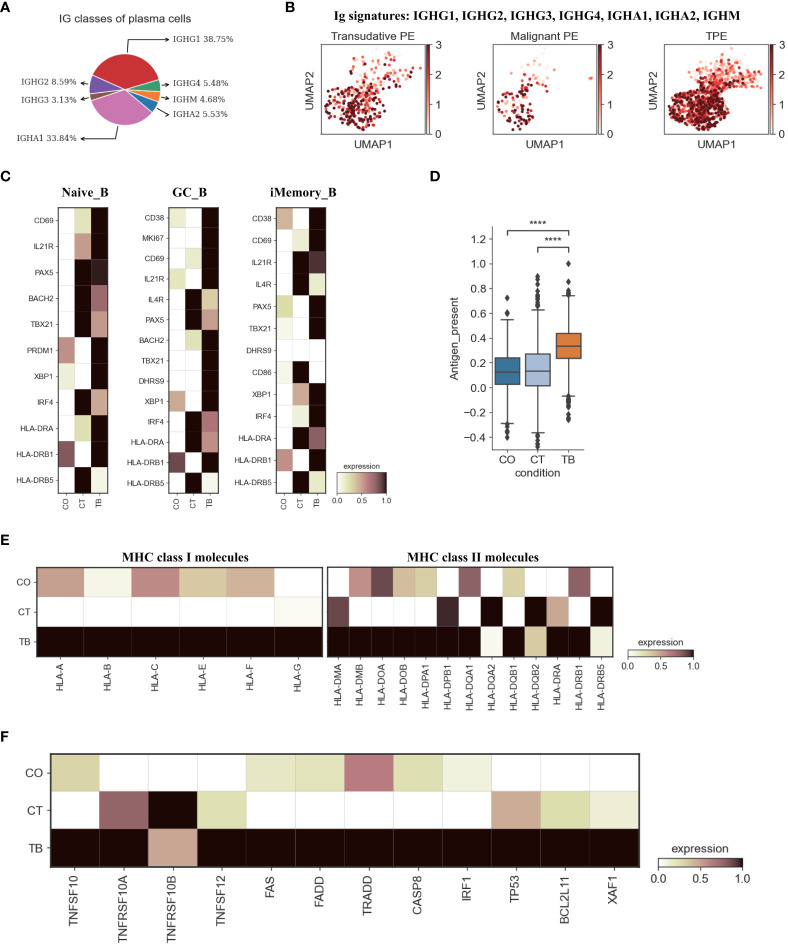
Characterization of gene expression differences in B cells across three conditions. **(A)** Proportion of heavy chain classes identified in plasma cells. **(B)** UMAP plots of mean gene expression from Ig signature genes in plasma cells, split by condition. **(C)** Heatmap showing the expression of selected B cell activation-associated genes in Naïve_B, GC_B and iMemory_B cell subset per condition. **(D)** Box plot showing the antigen presentation scores in B cells across three conditions. **(E)** Heatmap showing expression of HLA-I and HLA-II molecules in B cells across three conditions. **(F)** Heatmap of the expression of apoptosis-related genes in B cells across three conditions. Student’s T-test was applied to test significance in **(D)** ****p<0.0001.

Next, we examined the transcriptomic changes of B and PB cells (**Figures 5**, **S13**) in TPE, TSPE and MPE. Plasma cells highly expressed genes encoding the constant regions of immunoglobulin G1 (IgG1), IGHA1, IGHG2, IGHG4 and IGHM (**Figure 5A**), indicating their function in the secretion of antigen-specific antibodies. Plasma cells from TPE had a higher expression of Ig signature genes (IGHG1, IGHG2, IGHG3, IGHG4, IGHA1, IGHA2, IGHM) than TSPE and TPE (**Figure 5B**). Naïve_B from TPE were also enriched with key activation genes (e.g., CD69, IL21R, PAX5, BACH2 and HLA-DRA, etc.) (**Figure 5C**). Likewise, GC_B and iMemory_B also highly expressed their activation markers in TPE (GC_B for CD69, MKI67, HLA-DRB1, BACH2, and iMemory_B for TBX21, XBP1, IRF4, HLA-DRA) (**Figure 5C**). These results suggest that B/plasma cell-activation-associated pathways, such as somatic hypermutation, class switching, expansion and antibody production, were enriched in patients with TPE, implying that B/plasma cells from TPE may be activated for immune response to TB.

Previous reports have documented that B cell cytokines play an important in modulating T cell responses against intracellular bacteria while this has not been investigated in *Mtb* infection (27). We examined the key genes encoding representative cytokines in B cells, which are involved in T cell differentiation, expansion, and anti-*Mtb* response. Our data indicate that B cells from TPE were enriched with IL6, IL10, IL-12A and TNF (**Figure S14B**). Additionally, B cells are able to capture and internalize antigens via surface immunoglobulins, and then present these antigens on their surface as MHC II:peptide complexes to CD4 cells (especially to prime naïve CD4 T cells) (28). Thus, we analyzed the antigen presentation capacity of B cells in PF, and found that all B cell subsets from TPE displayed significantly higher antigen presenting capacity than transudative and malignant PE (**Figures 5D**, **S14C**). B cells from TPE highly expressed various presentation-associated genes (e.g., LGMN, CIITA, PFX5, TAP2, PSME1, TAP1, etc.) (**Figure S14D**). MHC molecules, especially MHC II, were significantly upregulated in B cells from TPE relative to TSPE and MPE (**Figure 5E**). These findings indicate that B cells from TPE might contribute to protection against *Mtb*-infection. In addition, we observed that apoptosis-associated genes (e.g. FAS, XAF1, TNFSF10, etc.) were upregulated in TPE compared to TSPE and MPE, implying that B cells in TPE likely underwent apoptosis (**Figure 5F**).

### Macrophages are the main drives of inflammation in TPE

**Figure 6:**
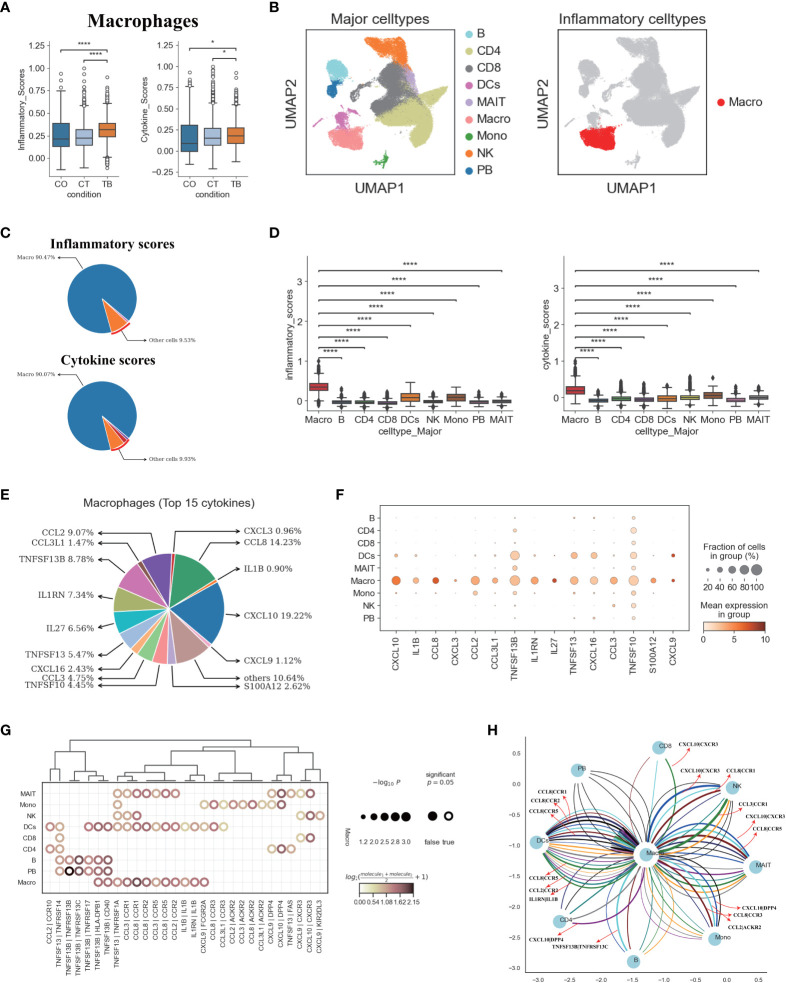
Macrophages in TPE as key cellular sources for inflammatory cytokines **(A)** Box plots of the inflammatory and cytokine scores in macrophage across three conditions. Student’s T-test was applied to test significance. *p<0.05, ****p<0.0001. **(B)** UMAP plots of PFMCs colored by: major cell types (Left panel) and inflammatory cell type (Right panel). **(C)** Pie charts showing the relative percentage contribution of each cell type to the inflammatory score (Top panel) and cytokine score (Bottom panel). **(D)** Box plots of inflammatory scores (Left panel) and cytokine scores (Right panel) in nine major cell types. **(E)** Pie charts showing the relative percentage contribution of each pro-inflammatory cytokine in macrophages from TPE. **(F)** Dot plot showing the expression of selected pro-inflammatory cytokines in TPE across nine major cell types. **(G)** Dot plot of the interactions between macrophages and other immune cell types in patients with TPE. P values are indicated by the circle sizes, as shown in the scale on the right. **(H)** Macrophage-other immune cell interaction network in patients with TPE. Interactions with P values <0.05 are shown. Representative ligand-receptor interactions between macrophages and other immune cell types are marked.

We next explored the potential sources of cytokine production in TPE. Using the reported inflammatory response genes and cytokine genes (**Table S9**) (15), we defined an inflammatory score and a cytokine score. Both scores were then used to assess the potential contribution to inflammation for each cell type. Macrophages were identified with significantly higher inflammatory and cytokine scores based on our scRNA-seq data from TPE samples (**Figures 6A, B**, **S15A**). This suggests that macrophages might be major sources of inflammation in TPE. Although the percentage of macrophages only reached ~ 3% in TPE (**Figure 1E**), the inflammatory and cytokine scores of macrophages reached 90% in TPE (**Figure 6C**) and were significantly higher than other cells (**Figure 6D**), further validating this cell type as inflammatory cells. In addition, five cell types, including B, CD8, MAIT, PB and NK cells, had higher inflammatory scores in TPE but their cytokine scores showed no difference compared to TSPE and MPE (**Figure S15A**). This suggest that these cell types may also contribute to inflammation response in TPE.

We then investigated the inflammatory signatures for pro-inflammatory macrophages in TPE. Our scRNA-seq data showed that macrophages in TPE had high expression of various pro-inflammatory cytokines (e.g., CCL2, CCL3, CXCL3, CCL8, IL1B, etc.) (**Figures 6E, F**, **S15B**) indicating various mechanisms leading to inflammation. The top 15 most highly expressed proinflammatory cytokines contributed to ~90% of the cytokine score (**Figure 6D**), highlighting the central role of these cytokines in driving inflammation in TPE (**Figures 6E**, **S15B**). Macrophages from TPE expressed significantly higher levels of these top 15 cytokines relative to other cells (e.g., B, CD4, CD8, NKs, etc.), further confirming their role as the major contributors to inflammation in TPE (**Figure S15B**). In addition, we observed significant elevated expression of inflammatory and cytokine genes in macrophages from TPE relative to TSPE and TPE (**Figures S15C, D**). Taken together, these findings illustrate that macrophages-driven inflammatory might be a distinct feature of TPE.

We reasoned that the systematic inflammatory response in patients with TPE may be related to the cross-talk between macrophages and other cells via secreting diverse pro-inflammatory cytokines such as those identified in the top 15. To investigate this, we examined the ligand-receptor pairing patterns between the hyper-inflammatory cell type (macrophages) and non-inflammatory cell types in TPE samples (**Figures 6G, H**). The interactions between macrophages and other cells appeared to display significant alterations (**Figure 6G**). Macrophages in TPE exhibited stronger interactions with DCs, monocytes and MAIT cells (**Figure 6G**). The interactions of macrophages with other cells mainly relied on CCR1, CCR2, CCR5, CCR3, CXCR3 and ACKR2 (**Figures 6G, H**). Interestingly, DCs cells in TPE expressed CCR1, CCR5 and CCR3, which can receive multiple cytokine stimuli generated by macrophages. Likewise, MAIT cells expressed CCR1, CCR5, CXCR3 and DPP4 while monocytes expressed CCR3, ACKR2 and DPP4, which can also receive multiple cytokine stimuli yielded from macrophages (**Figures 6G, H**). Furthermore, we observed that macrophages in TPE also could interact with itself using CCR1, CCR2, CCR5 and IL1B (**Figures 6G, H**). Taken together, these results illustrate the molecular basis for potential cell-cell interactions in TPE at the local site of infection in TB patients.

## Discussion

Tuberculosis (TB) caused by *Mtb* infection continues to be a severe threat to human health. Therefore, it is important to understand disease mechanisms, including mechanisms orchestrating local immune responses to *Mtb*, to effectively control this disease globally. Due to a lack of comprehensive data about the immune landscape in tissues, our understanding of disease mechanisms in TB is limited. The use of TPE is advantageous as it reflects the localized immune response to TB. Therefore, this study was the first to map the entire immune landscape, comprising of T-cells, NK cells, B cells and myeloid cells, to dissect the potential immune responses related to TPE and determine the potential sources of the inflammation in TPE.

By analyzing 78900 cells from TPE, MPE and TSPE, we identified 9 major cell-types and 37 subtypes, covering various immune cells in PF (**Figures 1**, **S1–S4**). Thus, this information-rich data enabled reliable analysis of these cell types or subtypes at different resolution. The proportion of different immune cells in PF were successfully defined and the compositional change for each was determined. Notably, various myeloid clusters, including DCs, monocytes and macrophages, were more enriched in MPE than TPE and TSPE (**Figure 1E**) suggesting that this might be a distinct characteristic of MPE and may be used as valuable biomarkers for differentiating MPE and TPE. In contrast, CD4^+^T and CD8^+^T cells were significantly increased in TPE relative to MPE, which can be used as other biomarkers to further differentiate MPE and TPE (**Figure 1E**). Additionally, CD4^+^T, B and PB cells were more enriched in TPE than TSPE while NK cells were significantly decreased (**Figure 1E**). These changes may be a promising biomarker for differentiating TPE and TSPE. Taken together, our scRNA-Seq data suggest that the relative abundance of immune cells in PF could be valuable for diagnosing TPE, and differentiating TPE from MPE and TSPE.

For T cells, our analysis suggested a high level of heterogeneity within T cell compartments among PFs. Previous reports have demonstrated that the T cell population in PF from TPE supports a Th1 response, with high levels of IFN-γ (29, 30). In our report, we identified two Th1 subtypes, including CD2_Th1-01 (immature Th1 cell) and CD4_Th1-02 (mature Th1 cell). TPE had a significantly higher proportion of CD4-Th1-02 cells than MPE and TSPE, which might be consistent with a phenomenon called “compartmentalization”, resulting in the paucibacillary nature in TPE and low yield in *Mtb* culture (11, 17). We found the two Th1 subtypes showed markedly higher IFNG expression in TPE compared to MPE and TSPE. This suggests higher production of IFN-γ in TPE. IFN-γ is required to activate macrophages and kill *Mtb* by promoting phagosomal maturation and production of reactive oxygen and nitrogen intermediates (26). In addition to the high expression of IFNG, the Th1 subtypes in TPE also displayed higher Th1 signatures (e.g., TBX21, GNLY, CXCR3, CD38, LTA, etc.), suggesting a Th1 response in TPE that is consistent with previous studies (29, 30). Moreover, we found that the two Th1 subtypes also highly expressed activation genes (e.g., CD69) and cytotoxic genes (e.g., GZMA, GZMK), suggesting that these two subtypes are likely multifunctional. In addition to the Th1 response, our data also supports a Th17 response in TPE, which may be related to protective immunity against TB.

We also identified two effector CD4 sub-clusters (CD4_eMemory-GZMA and CD4_eMemory-GZMK) in PF (including TPE) that shared similar gene expression characteristics with effector CD8 T sub-clusters (GZMA, GZMK, GZMM, KLRB1). These CD4 sub-clusters have not previously been identified in TPE. It has been hypothesized that these CD4 T effector clusters (especially effector memory CD4 T cells), possibly produced through repeated antigen stimulation, might play an important protective role against infectious diseases (e.g., *Mtb*) (31, 32). Although effector CD4 cells have been thought to employ various mechanisms to kill their target cells (33), the exact molecular mechanisms and their role in anti-*Mtb* remains unclear. Therefore, further studies should examine what role these effector CD4 T cells play in TB.

Growing evidence indicates that CD8 T cells play key roles in preventing and controlling *Mtb* infection through various granzymes (34, 35). Previous reports suggested that granzymes (e.g., GZMB) were able to directly kill *Mtb* in the presence of granulysin, via various mechanisms (36). In our report, we found that different CD8 T subclusters in TPE exhibited different phenotypes from those seen in MPE and TSPE. Effector CD8 T cells from TPE had the lowest cytotoxicity score and lower expression of cytotoxicity-related genes relative to TSPE and MPE. This suggests that effector CD8 T cell subclusters in TPE may have limited roles in anti-*Mtb*. Previous studies have demonstrated that low T cell responses are related to cell exhaustion and apoptosis (37, 38). Consistently, we found that XAF1 and TNF pathways were involved in CD8 T cell apoptosis in TPE, especially for effector CD8 T cells. Genes associated with the XAF1 and TNF pathways displayed higher expression in CD8 T cells from TPE than those from TSPE and MPE, potentially contributing to the low effector CD8 T cell response in TPE.

NK cells are recruited early to the site of infection and have an important role in amplifying the antimicrobial immune response to TB. By PAGA analysis, we confirmed that NK_Pro, as a proliferative subcluster, was an intermediate state. NK_Pro was connected to all other NK subtypes, indicating that this subtype might be valuable for therapeutic strategies targeting this intermediate NK subcluster. Unexpected, a dysfunctional NK response was found in TPE relative to TSPE and MPE, evidenced by low expression of cytokines and cytotoxicity-related genes. Our further analysis suggested that NK cell exhaustion and apoptosis may be the potential reason for the dysfunctional NK response in TPE. NK cells in TPE had high expression of multiple inhibitory receptors (e.g., PCDC1, LAG3, TIGIT, etc.). Similar to CD8 T cells, NK cells in TPE also showed a high apoptotic state, as genes associated with the XAF1, TNF and FAS pathways were upregulated in NK cells from TPE. These factors may result in functional impairment of NK cells in TPE.

Myeloid cells are an important component of the innate immune system for controlling and preventing *Mtb*. Myeloid cells including DCs, macrophages and monocytes in TPE showed stronger functional capacity for phagocytosis, antigen presentation and IFN-γ response as well as higher expression of HLA molecules, illustrating their effective role in anti-*Mtb*. Phagocytosis of *Mtb* by macrophages results in the formation of the phagosome and through a series of vesicle trafficking events, Mtb antigens are distributed through antigen processing and presentation pathways (25). Antigen-loaded MHC class II molecules are then shuttled to the plasma membrane to activate T cells and adaptive immune response against *Mtb*. In addition to macrophages, DCs also connect the adaptive and innate immune response through their role in capturing, processing and presenting antigens. Our findings show that macrophages and DCs in TPE had higher expression of HLA molecules and genes associated with phagocytosis and antigen presentation relative to TSPE and MPE, implying that these myeloid cells might provide the protective response to *Mtb* at the local site of infection. Interestingly, increased apoptosis of macrophages was also observed in TPE which may promote the release of *Mtb* antigen carrying vesicles. These vesicles can then be taken up by nearby DCs resulting in cross priming to further induce a protective response against *Mtb*. Additionally, our study observed that “response to IFN-γ” pathway in macrophages was significantly upregulated. IFN-γ is important for activating macrophages to kill engulfed *Mtb* via various mechanisms such as phagosome-lysosome fusion and generation of reactive oxygen and nitrogen intermediates (25, 39). This data indicate that myeloid cells in TPE may generate a protective response against *Mtb*.

Increasing evidence indicate that B cells and humoral immunity can modulate the immune response to various intracellular microbes, including *Mtb*, by producing cytokines and affecting T cell responses (40). B cells can present antigens to T cells with high efficiency by capturing and internalizing antigens via surface immunoglobulins. These antigens are then processed and presented on the surface as peptide:MHC class II complexes. Interestingly, B cells from TPE showed stronger antigen presenting capacity and had higher MHC II expression than B cells from TSPE and MPE, thus supporting their role as APCs that can prime T cells in TPE. We found that B cells from TPE might be activated due to the high expression of various activation genes. In addition, we observed that B cells in TPE highly expressed a wide variety of cytokines like IL6, IL10, IL-12A and TNF, which might influence the development of T cell-mediated immune response to *Mtb*. However, the detailed functions of B cell-derived cytokines in TPE remain to be evaluated. Our observations from TPE suggest that B cells may modulate the local immune response to *Mtb*.

In our attempt to explore the cellular origins of potential inflammatory cytokines, our data suggests that macrophages might be the major sources for these cytokines in TPE. This cell cluster might contribute to pro-inflammatory reaction via enhanced expression of pro-inflammatory cytokines such as CXCL10, CCL8, CCL2, TNFSF13B, IL1RN, CCL3, TNFSF13, etc. We found that hyper-inflammatory macrophages expressed multiple pro-inflammatory cytokines, highlighting potentially different mechanisms leading to the pro-inflammatory response in patients with TPE. In addition, potential cross-talk between macrophages and other cells were identified from our scRNA-seq data, as shown in **Figures 6G, H**. Targeting this crosstalk could be a potential strategy for controlling inflammation in future studies.

## Conclusion

Our comprehensive scRNA-seq dataset which covered three PFs (TPE, TSPE and MPE) revealed unique immune features in TPE that were not previously adequately appreciated. This data offers an important resource and crucial insights in revealing the localized immune response to TPE and potentially assist in the development of new effective therapeutics against *Mtb* infection.

## Methods

### Study design and participants

Adults with pleural effusion were prospectively recruited and sampled at Beijing Chest Hospital (Beijing, China). Enrolled participants had been administered anti-TB drug for ≤3 days in the past 6 months, had a detailed medical record and presented a minimum of 50 mL pleural fluid volume. According to Light’s criteria (41), pleural effusion was divided into exudative and transudative. For pleural TB cases, the inclusion criteria were: (1) Bacteriological evidence provided by culture, Xpert or PCR from pleural effusion; or (2) diagnosed as active pleural TB by a physician according to clinical findings, thoracoscopic reports and radiologic imaging. The exclusion criteria were: (1) had malignant tumors; (2) undergoing immunosuppressive therapy; (3) pregnant.

### Sample collection

**Supplementary Table 1** summarizes the characteristics of participants included in our study. Fresh PF samples from 2 patients with TSPE, 2 patients with MPE and 6 patients with TPE were immediately subjected to PFMCs isolation using standard density gradient centrifugation. Cell viability was measured using the Countstar cell viability detection kit. The cell viability was >90% for each sample and thus underwent cell encapsulation to generate 5’ gene expression profiles. Amplified cDNA was generated using a commercial emulsion-based microfluidic platform (Chromium 10x) and this cDNA was used for to prepare the single cell RNA-seq libraries.

### Single cell RNA library preparation and sequencing

The single cell RNA library preparation and sequencing was performed by NoveIBio Co., Ltd. (Shanghai) and as described in our previous studies (15, 42).

### Quantification and Statistical analysis

#### Single-cell RNA-seq data analysis

Single cell RNA-seq data was processed as previously described (15, 42). Briefly, the kallisto/bustools (kb v0.24.4) pipeline was used to generate the raw and filtered gene expression matrices. The anndata (ad) (v0.7.6) and scanpy (sc) (v1.7.2) packages in python (v3.8.10) were then used to analyze the filtered feature, barcode and matrix files. Potential doublets and low-quality cells were filtered and gene expression matrix were then normalized by library size to 10,000 reads per cell as described in Wang et al. (4, 42). The sc.pp.highly_variable_genes function was used to select the consensus set of 1,500 most highly-variable genes (HVGs) and prioritize gene features in the data with high cell-to-cell variations as previously described (43).

### Immune cell clustering and annotations

The sc.tl.louvain function was used to perform unsupervised clustering of cells at different resolutions. Using the neighborhood relations of cells, clustering consisted of two rounds: the first round (Louvain resolution = 2.0) identified 9 major cell types (CD4^+^ T cells, CD8^+^ T cells, MAIT cells, NK cells, B cells, plasma B cells, monocyte cells, dendritic cells, and macrophages) while the second round (with Louvain resolution 2.0) subdivided CD4+/CD8+ T, B, NK and DC cells into sub-clusters which represented distinct immune cell lineages within each major cell type. Each subset was confirmed by 1) manually matching canonical marker genes and 2) matching subset-specific signature genes using the sc.tl.rank_genes_groups function. Cluster annotation was also performed by manually matching canonical cell marker genes with subset-specific signature genes. Canonical marker genes and subset-specific signatures genes are provided in the main text and supplementary tables (**Tables S2–S7**).

### Cell state scores for immune cell subtypes

Defined gene sets for the were used to define and compare the overall activation level/physiological activity of cell clusters. The inflammatory response, pro-inflammatory cytokine and exhaustion response gene sets were collected from previous studies (15, 42). The leukocyte migration gene set (GO:0050900) and response to interferon-gamma (GO:0034341) were collected from MsigDB and previous studies (44–46). The cytotoxicity score was defined using 17 cytotoxicity-associated genes (*PRF1*, *IFNG*, *GNLY*, *NKG7*, *GZMA*, *GZMB*, *GZMH*, *GZMK*, *GZMM*, *KLRK1*, *KLRB1*, *KLRD1*, *FCGR3A*, *FGFBP2*, *ZEB2*, *CTSW* and *CST7*). The phagocytosis score was defined using 25 phagocytosis-related genes (ARF6, CDC42, ARPC4, PIK3R2, WASF2, ARPC1A, ARPC2, MARCKSL1, RAC2, CFL1, RPS6KB2, PRKCE, MARCKS, VAV2, DNM2, PIK3CG, FCGR3A, VASP, ARPC3, HCK, LYN, DOCK2, PLCG2, ARPC5, PTPRC). The antigen presentation score was defined using 36 antigen presentation-related genes (LGMN, CIITA, HLA-DMB, RFX5, HLA-DMA, NFYC, CTSL, IFI30, B2M, HLA-E, TAP2, PSME1, PSME2, HLA-F, HLA-C, HSP90AB1, HSPA8, HLA-DOA, CD74, HLA-DQA2, HLA-DQB1, HLA-DRA, HLA-DRB1, HLA-DRB5, HLA-DPA1, HLA-DQA1, HLA-DPB1, HSPA4, CALR, HSP90AA1, HLA-A, PDIA3, CTSB, PSME3, HLA-B, TAP1, CD4). The exhaustion response score and exhaustion score were defined using exhaustion response genes and exhaustion markers, respectively (**Table S8**). The migration score was defined using LEUKOCYTE MIGRATION Pathway (GO:0050900).The Scanpy sc.tl.score_genes function was used to calculate the cell state scores, which was defined as the average expression of genes from these predefined gene sets with respect to the reference genes. Comparison of the cell state scores between different groups were statistically assessed using t-test.

### Statistics

Statistical analysis and visualizations were performed in python and R and are provided with the results in the main text, in the figure legends or in the above Methods sections. The following symbols are used to indicate statistical significance for all figures: ns: p > 0.05; *p <= 0.05; **p <= 0.01; ***p <= 0.001; ****p <= 0.0001

### Code availability

Experimental protocols and pipelines used in this study follow the 10X Genomics and Scanpy official websites. Analysis steps, functions and parameters are described in detail in the Methods section. Custom scripts used to analyze data are available upon reasonable request.

### Software and algorithms

**Table:** 

Software	SOURCE	IDENTIFIER
**annadata**	pypi	https://github.com/theislab/anndata
**CellRanger v6.1.1**	10x Genomics	http://10xgenomics.com
**ggplot**	bioconductor	https://ggplot2.tidyverse.org
**ggpubr**	bioconductor	https://github.com/kassambara/ggpubr
**gseapy-0.10.7**	pypi	https://pypi.org/project/gseapy
**harmonypy**	pypi	https://github.com/slowkow/harmonypy
**kallistobustools-0.24.4**	pypi	https://github.com/pachterlab/kb_python Modular, efficient and constant-memory single-cell RNA-seq preprocessing. *Nat Biotechnol* 39, 813–818 (2021).
**scanpy v1.7.2**	bioconda	https://github.com/theislab/scanpy
**scirpy v0.7.0**	bioconda	https://github.com/icbi-lab/scirpy
**scrublet v0.2.3**	pypi	https://github.com/swolock/scrublet
**statannot**	pypi	https://pypi.org/project/statannot

## Data availability statement

The data presented in the study are deposited in the OMIX repository, accession number OMIX004145.

## Ethics statement

Ethics approval for this study was obtained from the Beijing Chest Hospital ethics committee (ethical approval No. YNLX-2022-005). Written informed consent was acquired from each participant. The patients/participants provided their written informed consent to participate in this study.

## Author contributions

YW conceived the study. YW and GW designed the study. YW, GW, JP and LL supervised this project. XY, JY, YX, QS, YZ, RG, CHW, XuL, QL, HW, CW, XiL, SL, MZ, RW, HZ, YL and NC performed the experiments. XY, YW, GW and JP contributed the reagents, materials, and analysis tools. YW performed the software. YW, XY, GW and LL analyze the data; YW drafted the original paper. YW, LL revised and edited this paper. LL, JP, GW and YW reviewed the paper. All authors read and approved the final manuscript.
