# Supplementary material for: Single-cell profiling reveals distinct immune response landscapes in tuberculous pleural effusion and non-TPE
Source: Front Immunol. 2023 Jun 26;14:1191357. doi: 10.3389/fimmu.2023.1191357 (PMC10331301; doi:10.3389/fimmu.2023.1191357)
Supplement: Supplementary file 16 [file DataSheet_1.docx]

**Supplementary**

**Single-cell profiling reveals immune response landscapes in tuberculous pleural effusion**

Xinting Yang ^1,*^, Jun Yan ^2,*^, Yu Xue^3,*^, Qing Sun ^4^, Yun Zhang ^1^, Ru Guo ^1^, Chaohong Wang ^2^, Xuelian Li^1^, Qingtao Liang ^1^, Hangyu Wu ^5^, Chong Wang^5^, Xinlei Liao^2^, Sibo Long^2^, Maike Zheng^2^, Rongrong Wei^6^, Haoran Zhang^6^, Yi Liu^6^, Nanying Che^6^, Laurence Don Wai Luu ^7,†^, Junhua Pan^8,†^ , Guirong Wang ^2,†^, Yi Wang ^9,†^

^1^ Tuberculosis Department, Beijing Chest Hospital, Capital Medical University, Beijing, 101149, P.R. China

^2^ Department of Clinical Laboratory, Beijing Chest Hospital, Capital Medical University, Beijing Tuberculosis and Thoracic Tumor Institute, Beijing, 101149, P.R. China

^3^ Department of Emergency, Beijing Chest Hospital, Capital Medical University, Beijing, 101149, P.R. China

^4^ National Clinical Laboratory on Tuberculosis, Beijing Key Laboratory for Drug-Resistant Tuberculosis Research, Beijing Chest Hospital, Capital Medical University, Beijing Tuberculosis and Thoracic Tumor Institute, Beijing, 101149, P.R. China

^5^ Heart Center, Beijing Chest Hospital, Capital Medical University, Beijing, 101149, P.R. China

^6^ Biobank, Beijing Chest Hospital, Capital Medical University, Beijing, 101149, P.R. China

^7^ School of Life Sciences, University of Technology Sydney, New South Wales, 2007, Australia

^8^ Beijing Chest Hospital, Capital Medical University, Beijing, 101149, P.R. China

^9^ Experimental Research Center, Capital Institute of Pediatrics, Beijing, 100020, P.R. China.

^†^ Correspondence:

Prof. **Yi Wang**, [wildwolf0101@163.com](mailto:wildwolf0101@163.com) (Handing the correspondence)

Prof. **Guirong Wang**, [wangguirong1230@ccmu.edu.cn](mailto:wangguirong1230@ccmu.edu.cn)

Prof. **Junhua Pan**, [panjunhua999@sina.com](mailto:panjunhua999@sina.com)

Prof. **Zhenjun Li**, [lizhenjun@icdc.cn](mailto:lizhenjun@icdc.cn)

Dr. **Laurence Don Wai Luu**, [laurence.luu@uts.edu.au](mailto:laurence.luu@uts.edu.au)

**^*^** These authors contributed equally to this article

**Figure Legends**

**Fig S1. Characteristics of the integrated dataset collected from 2 patients with TSPE, 2 patients** **with MPE and 6 patients with TPE.**

A, Distribution of unique molecular identifier (UMI) counts per cell detected in various conditions.

B, Distribution of unique molecular identifier (UMI) counts per cell detected in each sample.

C, Distribution of gene counts per cell detected in various conditions.

D, Distribution of gene counts per cell detected in each sample.

E**,** Percentage of mitochondrial transcripts per cell detected in various conditions.

F**,** Percentage of mitochondrial transcripts per cell detected in each sample.

**Fig S2. Characteristics of selected markers for cell type/subtype in different cell lineages, related to Figure 1.**

A. Dot plots of selected marker genes (Rows) for the 9 major cell types (Columns).

B-F. Dot plots of selected marker genes (Rows) for each cell subtype (Columns), including 9 CD4^+^T cell subtypes (B), 9 CD8^+^T cell subtypes (C), 6 NK cell subtypes (D), 4 DCs subtypes (D) and.6 B/plasma B cell subtypes (F).

**Fig S3 The clustering result for CD4^+^T, CD8^+^T, NK, DCs and B/plasma.**

A-F. The clustering result for CD4^+^T (*n*=9), CD8^+^T (*n*=8), NK (*n*=6), DCs (*n*=4) and B/plasma (*n*=6) cell subsets. Each point represents one single cell, colored according to cell type.

**Fig S4. Detailed data output and visualization of major cell types identified in single-cell transcriptional profiling of PFMCs from 10 samples, related to figure 1.**

A. The UMAP projection of the 9 major cell type in each of the three conditions. Cells are colored by the 9 major cell types.

B. Relative proportion of the 9 cell subtypes derived from CO, CT and TB conditions.

C. The UMAP projection of the 10 individual samples. Cells are colored according to each individual.

D. Stack boxplots showing the relative proportion of the 9 major cell types in each individual sample.

E. The composition of each immune cell type across 3 conditions. The y-axis shows the average percentage of each immune cell. Conditions are displayed in different colors on the *x* axis. Student’s T-test was applied to test the significance. *p<0.05, **p<0.01, ***p<0.001, ****p<0.0001, ^ns^p>0.05.

**Fig S5. Detailed data output and visualization of cell subtypes identified in single-cell transcriptional profiling of PFMCs from 10 samples, related to figure 1.**

A. The UMAP projection of the 37 cell subtypes in each of the three conditions. Cells are colored by the 37 cell subtypes.

B. Relative proportion of the 37 cell subtypes derived from CO, CT and TB conditions.

C. The UMAP projection of the 10 individual samples with different colors. Cells are colored according to the three conditions.

D. Stack boxplots showing the relative proportion of the 37 cell subtypes in each individual sample.

**Fig S6. Detailed data output and visualization of single-cell transcriptional profiling of T cells from 10 samples, related to figure 2.**

A. The clustering result of the 19 T cell subtypes from 10 individuals. Each point represents one single cell, colored according to T cell subtypes.

B. The UMAP projection of the 19 T cell subtypes across three conditions. Cells are colored by the 19 T cell subtypes.

C. Relative proportion of the 19 T cell subtypes derived from CO_,_ CT and TB conditions.

D. The UMAP projection highlighting the 10 individual samples with different colors. Cells are colored according to the 10 individuals.

E. Stack boxplots showing the relative proportion of the 19 T cell subtypes in each individual sample.

F. The distribution of each T cell subtype across 3 conditions. The y-axis shows the average percentage of each immune cell across the 3 conditions. Conditions are displayed in different colors on the *x* axis. Student’s T-test was applied to test the significance. *p<0.05, **p<0.01, ***p<0.001, ****p<0.0001, ^ns^p>0.05.

**Fig S7**. **Detailed data about T cell gene expression.**

A, Dot plots showing the expression of selected genes in each CD4^+^T cell subtype across conditions.

B, Dot plots showing the expression of selected genes in each CD8^+^T cell subtype across conditions.

C. UMAP plots of mean gene expression from T cell activation gene signatures, split by condition.

D. Box plots showing the TNF expression in CD4_Th1-01 and CD4_Th1-02 cells for each condition.

E. Box plots showing the cytotoxicity score in effector T cell subtypes for each condition.

F. Heatmap plots showing the cytotoxicity-related gene expression in T cells in the three conditions.

**Fig S8**. **Detailed data about T cell gene expression.**

A. Box plots showing exhaustion response scores and exhaustion scores in each effector T cell subtype for each condition.

B. Dot plots showing exhaustion marker expression in activated CD8^+^T and CD4^+^T cells.

C. Dot plots showing the expression of apoptosis-related genes in each CD4^+^T cell subset per condition.

C. Dot plots showing the expression of apoptosis-related genes in each CD8^+^T cell subset per condition.

**Fig S9. Detailed data output and visualization of single-cell transcriptional profiling of NK cells from 10 samples, related to figure 3.**

A. The clustering result for 6 NK cell types from all samples. Each point represents one single cell, colored according to NK cell subtype.

B. The UMAP projection of the 6 NK cell subtypes across three conditions . Cells are colored by the 6 NK cell subtypes.

C. Relative proportion of the 6 NK cell subtypes derived from CO_,_ CT and TB conditions.

D. The NK UMAP projection highlighting the 10 individual samples with different colors. Cells are colored according to the 10 individuals.

E. Stack boxplots showing the relative proportion of the 6 NK cell subtypes in each sample.

F. The distribution of each NK cell subtype across 3 conditions. The y-axis shows the average percentage of each NK subtype across the 3 conditions. Conditions are displayed in different colors on the *x* axis. Student’s T-test was applied to test the significance. *p<0.05, **p<0.01, ***p<0.001, ****p<0.0001, ^ns^p>0.05.

**Fig S10**. **Detailed data about NK cell gene expression.**

A. Dot plots showing expression of selected genes in NK cells per condition.

B. UMAP plots of mean gene expression from cytotoxic gene signatures in NK cells, split by condition.

C. Box plots showing exhaustion response score and exhaustion score in each effector NK cell subset across each condition.

D. Heatmap showing selected apoptosis-related gene expression in NK cells in the three conditions.

E. Box plots of leukocyte migration scores in NK cell subtypes across different conditions.

Student’s T-test was applied to test significance in C, D and E *p<0.05, **p<0.01, ***p<0.001, ****p<0.0001, ^ns^p>0.05.

**Fig S11. Detailed data output and visualization of single-cell transcriptional profiling of myeloid cells from 10 samples, related to figure 4.**

A. The clustering result of 6 myeloid cell subtypes from 10 individuals. Each point represents one single cell, colored according to myeloid cell subtype.

B. The UMAP projection of the 6 myeloid cell subtypes across three conditions. Cells are colored by the 6 myeloid cell subtypes.

C. Relative proportion of the 6 myeloid cell subtypes derived from CO, CT and TB conditions.

D. The myeloid cell UMAP projection highlighting the 10 individual samples with different colors. Cells are colored according to the 10 individuals.

E. Stack boxplots showing the relative proportion of the 6 myeloid cell subtypes in each sample.

F. The distribution of each myeloid cell subtype across 3 conditions. The y-axis shows the average percentage of each myeloid cell subtype across the 3 conditions. Conditions are displayed in different colors on the *x* axis. Student’s T-test was applied to test the significance. *p<0.05, **p<0.01, ***p<0.001, ****p<0.0001, ^ns^p>0.05.

**Fig S12**. **Detailed data about myeloid cell gene expression.**

A. Heatmap plots showing selected phagocytosis- and antigen presentation-associated gene expression in monocytes in the three conditions.

B. Heatmap plots showing selected phagocytosis-related gene expression in macrophages in the three conditions.

C. Heatmap plots showing selected antigen presentation- and HLA-II-related gene expression in macrophages in the three conditions.

D. Box plots showing the response to interferon-gamma score in macrophage per condition.

E. UMAP plots of mean gene expression from apoptosis gene signatures in macrophages, split by condition.

Student’s T-test was applied to test significance in D. *p<0.05, **p<0.01, ***p<0.001, ****p<0.0001, ^ns^p>0.05.

**Fig S13. Detailed data output and visualization of single-cell transcriptional profiling of B cells from 10 samples, related to figure 5.**

A. The clustering result of 6 B cell subtypes from 10 individuals. Each point represents one single cell, colored according to B cell subtype.

B. The UMAP projection of the 6 B cell subtypes across three conditions. Cells are colored by the 6 B cell subtypes.

C. Relative proportion of the 6 B cell subtypes derived from CO, CT and TB conditions.

D. The UMAP projection highlighting the 10 individual samples with different colors. Cells are colored according to the 10 individuals.

E. Stack boxplots showing the relative proportion of the 6 B cell subtypes in each sample.

F. The distribution of each B cell subtypes across 3 conditions. The y-axis shows the average percentage of each immune cell across the 3 conditions. Conditions are displayed in different colors on the *x* axis. Student’s T-test was applied to test the significance. *p<0.05, **p<0.01, ***p<0.001, ****p<0.0001, ^ns^p>0.05.

**Fig S14**. **Detailed data about B cell gene expression.**

A. PAGA analysis of B cell pseudo-time: the associated cell type is shown.

B. Heatmap showing the expression of selected genes in B cells in the three conditions.

C. Box plots showing the antigen presentation score in each B cell subtype per condition.

D. Heatmap showing selected antigen presentation-associated gene expression in B cells in the three conditions.

Student’s T-test was applied to test significance in C *p<0.05, **p<0.01, ***p<0.001, ****p<0.0001, ^ns^p>0.05.

**Fig S15. Detailed data about the pro-inflammatory response in patients with TPE.**

A. Box plots showing the inflammatory and cytokine scores for each major cell type across three conditions.

B. Box plots showing the expression of major pro-inflammatory genes across each major cell type.

C. Violin plots showing the inflammatory response scores (left panel (top)) and cytokine scores (left panel (bottom)) in macrophages across three conditions; UMAP plots of mean gene expression of the inflammatory response scores (right panel (top)) and cytokine scores (right panel (bottom)) in macrophages, split by condition.

D. Box plots showing the expression of major pro-inflammatory genes in macrophages across the three conditions.
