# Supplementary material for: Single-cell profiling reveals distinct immune response landscapes in tuberculous pleural effusion and non-TPE
Source: Front Immunol. 2023 Jun 26;14:1191357. doi: 10.3389/fimmu.2023.1191357 (PMC10331301; doi:10.3389/fimmu.2023.1191357)
Supplement: Supplementary file 17 [file Table_1.docx]

Supplementary Table 1. General characteristics and clinical features associated with participants

| Transudative pleural effusion (TSPE) | | | | | | |
| --- | --- | --- | --- | --- | --- | --- |
| Chest X-ray | 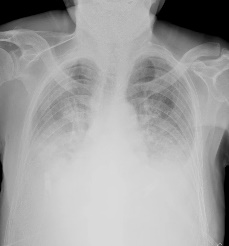 | 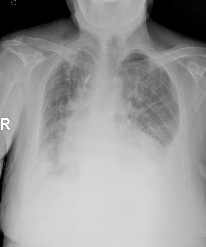 |  |  |  |  |
| Code | CO1 | CO2 |  |  |  |  |
| Gender | Male | Female |  |  |  |  |
| Age (years) | 79 | 81 |  |  |  |  |
| Diagnosis | Right ventricle dysfunction ，  Coronary atherosclerotic heart disease | Hypertrophic obstructive cardiomyopathy |  |  |  |  |
| Malignant pleural effusion (MPE) | | | | | | |
| Chest X-ray | 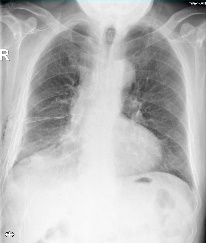 | 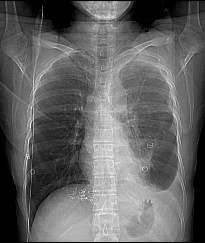 |  |  |  |  |
| Code | CT1 | CT2 |  |  |  |  |
| Gender | Male | Female |  |  |  |  |
| Age (years) | 69 | 41 |  |  |  |  |
| Diagnosis | Malignant pleural mesothelioma | Lung adenocarcinoma with pleural metastasis |  |  |  |  |
| Tuberculous pleural effusion (TPE) | | | | | | |
| Chest X-ray | 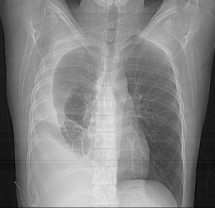 | 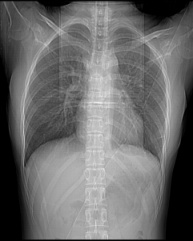 | 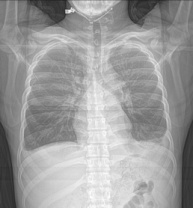 | 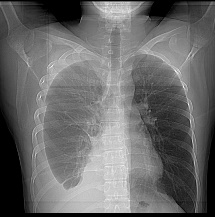 | 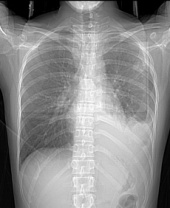 | 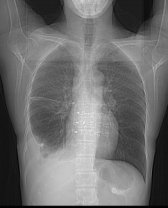 |
| Code | TB1 | TB2 | TB3 | TB4 | TB5 | TB6 |
| Gender | Male | Female | Male | Male | Female | Female |
| Age (years) | 19 | 19 | 22 | 20 | 32 | 57 |
| Clinical features | Exudates,  PCR(+),  ADA=55.1,  IGRAs(+), | Exudates,  PCR(+),  ADA=42,  IGRAs(+) | Exudates,  PCR(-),  ADA=32,  IGRAs(+) | Exudates,  PCR(+),  ADA=68.6,  IGRAs(+) | Exudates,  PCR(-),  ADA=31.8,  IGRAs(+) | Exudates,  PCR(+),  ADA=39.2,  IGRAs(+) |
| Diagnosis | Definite tuberculous pleurisy | Definite tuberculous pleurisy | Clinical tuberculous pleurisy | Definite tuberculous pleurisy | Clinical tuberculous pleurisy | Definite tuberculous pleurisy |
| Clinical outcome | Improvement | Improvement | Improvement | Improvement | Improvement | Improvement |
